# Secretagogin is Related to Insulin Secretion but Unrelated to Gestational Diabetes Mellitus Status in Pregnancy

**DOI:** 10.3390/jcm9072277

**Published:** 2020-07-17

**Authors:** Carola Deischinger, Jürgen Harreiter, Karoline Leitner, Dagmar Bancher-Todesca, Sabina Baumgartner-Parzer, Alexandra Kautzky-Willer

**Affiliations:** 1 Clinical Division of Endocrinology and Metabolism, Department of Internal Medicine III, Gender Medicine Unit, Medical University of Vienna, Waehringer Guertel 18–20, 1090 Vienna, Austria; carola.deischinger@meduniwien.ac.at (C.D.); karoline.leitner@meduniwien.ac.at (K.L.); sabina.baumgartner-parzer@meduniwien.ac.at (S.B.-P.); alexandra.kautzky-willer@meduniwien.ac.at (A.K.-W.); 2 Department of Obstetrics and Gynecology, Medical University of Vienna, Waehringer Guertel 18–20, 1090 Vienna, Austria; dagmar.bancher-todesca@meduniwien.ac.at

**Keywords:** biomarker, gestational diabetes mellitus, insulin secretion, pregnancy, secretagogin

## Abstract

Secretagogin (SCGN) is a calcium binding protein related to insulin release in the pancreas. Although SCGN is not co-released with insulin, plasma concentrations have been found to be increased in type 2 diabetes mellitus patients. Until now, no study on SCGN levels in pregnancy or patients with gestational diabetes mellitus (GDM) has been published. In 93 women of a high-risk population for GDM at the Medical University of Vienna, secretagogin levels of 45 GDM patients were compared to 48 women with a normal glucose tolerance (NGT). Glucose tolerance, insulin resistance and secretion were assessed with oral glucose tolerance tests (OGTT) between the 10th and 28th week of gestation (GW) and postpartum. In all women, however, predominantly in women with NGT, there was a significant positive correlation between SCGN levels and Stumvoll first (*r_p_* = 0.220, *p* = 0.032) and second phase index (*r_p_* = 0.224, *p* = 0.028). SCGN levels were not significantly different in women with NGT and GDM. However, SCGN was higher postpartum than during pregnancy (postpartum: 88.07 ± 35.63 pg/mL; pregnancy: 75.24 ± 37.90 pg/mL, *p* = 0.004). SCGN was directly correlated with week of gestation (*r_p_* = 0.308; *p* = 0.021) and triglycerides (*r_p_* = 0.276; *p* = 0.038) in women with GDM. Therefore, SCGN is related to insulin secretion and hyperinsulinemia during pregnancy; however, it does not display differences between women with NGT and GDM.

## 1. Introduction

Secretagogin (SCGN) is a calcium-binding protein, which was discovered in the pancreas in 1998 [1]. Many interactions of SCGN have not yet been fully understood; however, they are assumed to be of importance to the function of secretory cells [2,3,4,5,6,7]. Besides neuroendocrine cells such as the islets of Langerhans and developing or adult neurons, SCGN can be found in the thyroid, gastrointestinal tract, adrenal medulla, adrenal gland and brain [1]. Its involvement with vesicle trafficking and exocytosis has already been proven for insulin release in pancreatic islet cells and corticotropin-releasing hormone (CRH) mediated stress responses [8,9,10]. The exact mechanism of secretagogin’s influence on insulin release has only been understood rather recently and involves SCGN-interacting proteins which are either actin-binding proteins, involved in insulin granule trafficking and exocytosis [11] or have a regulatory function towards the actin cytoskeleton by facilitating vesicle transport to the periphery during insulin release [12]. SCGN further binds insulin and improves insulin signaling when compared to insulin action alone by increasing insulin-induced phosphorylation of Akt [13]. Accordingly, SCGN was recently found to be elevated in patients with type 2 diabetes [14] and has been related to beta-cell proliferation and insulin secretion [9]. In pregnancy, insulin resistance increases to ensure the glucose supply of the fetus, which is countered by a higher level of insulin production and secretion [15,16]. The pancreatic islets adapt to cope with the increased insulin production and secretion. Islet cell mass increases 1.4–2-fold during gestation [17,18], whereas adult islet cell mass remains steady and proliferates slowly outside of pregnancy [19]. If these adaptions are insufficient and the insulin supply fail to match tissue demand, pregnant women develop gestational diabetes mellitus (GDM) [20]. GDM is a form of hyperglycemia with its onset or first detection during pregnancy and has a prevalence of 2 to 6% in Europe [21]. Not only is GDM associated with an increased risk for complications for both mother and child during pregnancy and childbirth [22] but also have women who suffer from gestational diabetes a 3.44 elevated risk for developing type 2 diabetes mellitus postpartum [23]. Up to this day, no studies on SCGN and its possible roles during pregnancy have been published. Previously, SCGN was associated with beta-cell proliferation, insulin secretion and was increased in patients with diabetes mellitus. Therefore, we hypothesized SCGN might be similarly involved in GDM and aimed at investigating SCGN as a marker of insulin secretion in the context of pregnancy, postpartum and the development of GDM.

## 2. Materials and Methods

### 2.1. Study Participants and Design

The study population included 93 pregnant women (48 women with normal glucose tolerance (NGT), 45 with GDM) of all body mass index (BMI) categories recruited for two prospective longitudinal studies conducted at the Medical University of Vienna between 2010 and 2014. Both studies were approved by the local ethics committee (Ethics Committee of the Medical University of Vienna, EK Nr. 2022/2012 & 771/2008) and was performed in accordance with the Declaration of Helsinki. All subjects gave written informed consent for participation in the study [24]. Inclusion criteria were a singleton pregnancy and age ≥ 18 years. Exclusion criteria were pre-existing diabetes, chronic and/or infectious diseases, significant psychiatric disorders or inability to follow instructions related to the studies due to language difficulties. All study subjects were monitored and treated during their pregnancy following the national guidelines [25,26]. As a tertiary health care center taking care of higher risk pregnancies, a high number of cases with GDM is represented in our cohort. GDM was assessed according to IADPSG WHO guidelines [27]. Oral glucose tolerance tests and further clinical evaluations were performed at week of gestation (GW) 10–28 and postpartum (mean = 8 ± 6 months after delivery), respectively, with blood samples taken at baseline, 30, 60, 90 and 120 min for the measurement of glucose, insulin and c-peptide. Hemoglobin A1c according to the International Federation of Clinical Chemistry working group (HbA1c-IFCC) and levels of triglycerides, cholesterol, creatine and bioavailable estradiol were analyzed in our ISO 9001 certified central laboratory at the General Hospital in Vienna (AKH Wien, Austria, www.kimcl.at). Weight was measured on calibrated electronic scales (SECA 877/888, SECA, Hamburg, Germany) wearing no shoes and light clothes. Waist circumference was measured twice at the midpoint between the lower border of the rib cage and the iliac crest and hip circumference at the widest portion of the buttocks. Systolic and diastolic blood pressure and heart rate were measured on the left arm with an appropriate-sized cuff with an BOSO medicus device (Bosch + Sohn, Jungingen, Germany).

### 2.2. Calculation of Insulin Secretion and Sensitivity Indices

Approximations of insulin sensitivity and insulin secretion were calculated (Matsuda Index, Stumvoll first and second phase index, insulin secretion sensitivity index (ISSI-2), disposition index, area under the curve (AUC) insulin and glucose) for each oral glucose tolerance tests (OGTT). The Matsuda Index is an estimate of peripheral and hepatic insulin sensitivity (liver, muscle and adipose tissue). Due to the complexity of the formula, an online calculator was used [28]. Stumvoll first phase index for insulin secretion was calculated as 1.283 + 1.829 × Insulin 30min − 138.7 × Glucose 30min + 3.772 × Insulin 0 min for estimated first phase beta cell function. Stumvoll second phase index was calculated with the formula 287 + 0.4164 × Insulin 30 min − 26.07 × Glucose 30min + 0.9226 × Insulin 0 min [29]. Oral disposition index is the product of the Matsuda Index and Δ Insulin 0 − 30/Δ Glucose 0 − 30 [30,31]. To improve the assessment of beta-cell reserve, ISSI-2, the product of the Matsuda Index and the ratio of the area-under-the-insulin curve to the area-under-the-glucose curve, was used [32]. AUC insulin and AUC glucose were calculated using the trapezoidal method.

### 2.3. SCGN Assay

For the serum SCGN analysis, a human ELISA kit (BioVendor, Brno, Czech Republic) was used (https://www.biovendor.com/secretagogin-human-elisa?d=114). The detection range of this kit is 62.5–2000 pg/mL with an inter-assay coefficient of variability (CV) of 6.5% and an intra-assay CV of 6%. Samples were diluted 1 + 1 with dilution buffer (provided in the assay kit), internal control samples were analyzed in each assay and the measured concentrations were in the expected range. As internal controls gave almost identical results in three different assays adjustment for inter-assay bias was not done.

### 2.4. Statistical Analysis

Descriptive data analysis was performed for all parameters. Continuous variables were summarized by mean ± SD and categorical variables by counts and percentages. Assumption of Gaussian distribution of parameters was decided by visual assessment of histograms and calculation of skewness using Kolmogorov–Smirnov test. Consequently, the non-parametrically distributed parameter SCGN was log transformed. All women with SCGN values outside the reference range of the SCGN kit (62.5–2000 pg/mL) and outliers with 2 × 1,5 IQR were excluded from the analysis. An independent samples T-Test was used to investigate differences in SCGN levels between NGT and GDM in pregnancy. Due to missing values, postpartum SCGN values were not available for all women. In a subgroup of women with both pregnancy and postpartum SCGN values (*n* = 34), a paired T-test was performed. To assess SCGN levels over the course of an OGTT, a repeated measure ANOVA (with Greenhouse–Geisser correction due to rejected sphericity assumption) was calculated. Pearson’s correlation was used for a correlation analysis. As this is a post hoc analysis, a power analysis was omitted. For the statistical analysis, SPSS 25.0 (SPSS Inc, Chicago, USA) was used. A two-sided *p*-value ≤ 0.05 was considered statistically significant.

## 3. Results

### 3.1. Baseline Characteristics

Characteristics of the study population are presented in Table 1 and show significant differences between the groups in anthropometric, glycemic and metabolic parameters. The AUC insulin (*p* = 0.024) and AUC glucose (*p* < 0.001) were elevated in women with GDM compared to NGT. Matsuda index (*p* = 0.013), ISSI-2 (*p* < 0.001), disposition index (*p* < 0.001), Stumvoll first phase (*p* = 0.003) and second phase index (*p* = 0.020) were lower; HbA1c (*p* = 0.002) and triglycerides (*p* = 0.014) higher in GDM than in NGT. Postpartum, none of the glycemic and metabolic parameters differed between NGT and those who had had GDM during pregnancy. Fetal parameters such as fetal weight, length, abdominal and head circumference did not differ between the groups either.

### 3.2. SCGN Levels in NGT and GDM During Pregnancy and Postpartum

**Figure 1:**
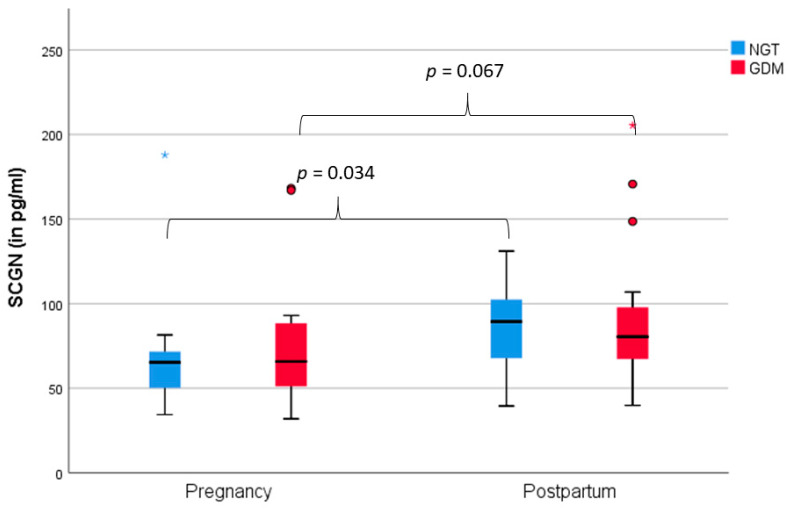
Clustered boxplot of secretagogin (SCGN) levels in normal glucose tolerance (NGT) (*n* = 19) and gestational diabetes mellitus (GDM) (*n* = 15) during pregnancy and postpartum.

In the whole cohort, SCGN levels were significantly lower during pregnancy (mean = 75.24 pg/mL, SD = 37.90 pg/mL) compared to postpartum (mean = 88.07 pg/mL, SD = 35.63 pg/mL, *p* = 0.004). As illustrated in Figure 1, SCGN levels were higher postpartum in NGT (*p* = 0.034). SCGN levels displayed the same trend in women who had had GDM during gestation, albeit not significant (*p* = 0.067).

Unlike in type 2 diabetes mellitus patients, there was no difference in SCGN levels between NGT and GDM (pregnancy: *p* = 0.514; postpartum: *p* = 0.683). When investigating SCGN over the course of an OGTT in a small subgroup of 9 women (5 NGT, 4 GDM), SCGN did not change significantly (*p* = 0.100) from 77.9 pg/mL (± 55.0 pg/mL) at baseline, 74.83 pg/mL (± 52.0 pg/mL) after 60 min to 80.1 pg/mL (± 52.5 pg/mL) at 120 min.

### 3.3. Correlation of SCGN with Covariates in Pregnancy and Postpartum

**Figure 2:**
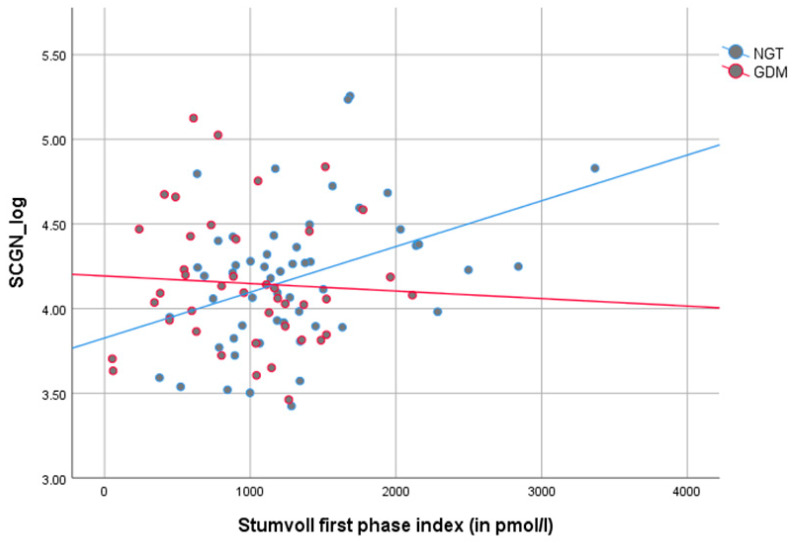
Grouped scatter dot plot according to glucose tolerance (blue = NGT, red = GDM) of the correlation between secretagogin (SCGN) (log-transformed) and Stumvoll first phase index during pregnancy. SCGN correlated directly with Stumvoll first phase index in women with NGT (*r_p_* = 0.390, *p* = 0.004), an effect which was not present in GDM (*r_p_* = −0.056, *p* = 0.724). NGT: normal glucose tolerance; GDM: gestational diabetes mellitus.

**Figure 3:**
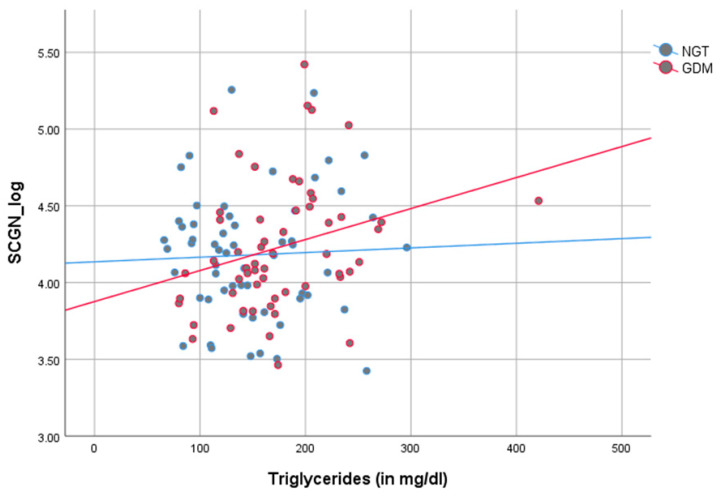
Grouped scatter dot plot according to glucose tolerance (blue = NGT, red = GDM) of the correlation between secretagogin (SCGN) (log-transformed) triglycerides during pregnancy. SCGN correlated positively with triglycerides (TG) in women with GDM (*r_p_* = 0.276, *p* = 0.038). In NGT, a similar trend, albeit not significant, is visible (*r_p_* = 0.041, *p* = 0.761). NGT: normal glucose tolerance; GDM: gestational diabetes mellitus; TG: triglycerides.

As demonstrated in Figure 2 and Table 2, there was a direct correlation between SCGN levels and Stumvoll first (*r_p_* = 0.220, *p* = 0.032) and second phase index (*r_p_* = 0.224, *p* = 0.028), parameters of insulin secretion, in all women during pregnancy, however, predominantly in women with NGT. In women with NGT, SCGN correlated positively with Stumvoll first (*r_p_* = 0.390, *p* = 0.004) and second phase index (*r_p_* = 0.395, *p* = 0.003), AUC insulin (*r_p_* = 0.380, *p* = 0.005) and HbA1c (*r_p_* = − 0.391, *p* = 0.002) and negatively with the Matsuda index (*r_p_* = − 0.273, *p* = 0.050). Postpartum, these glycemic indices ceased to correlate with SCGN. Furthermore, SCGN was directly correlated with creatine (*r_p_* = 0.194, *p* = 0.012) in the whole cohort and with triglycerides (*r_p_* = 0.276, *p* = 0.038) in women with GDM during pregnancy (see Figure 3 and Table 2).

**Figure 4:**
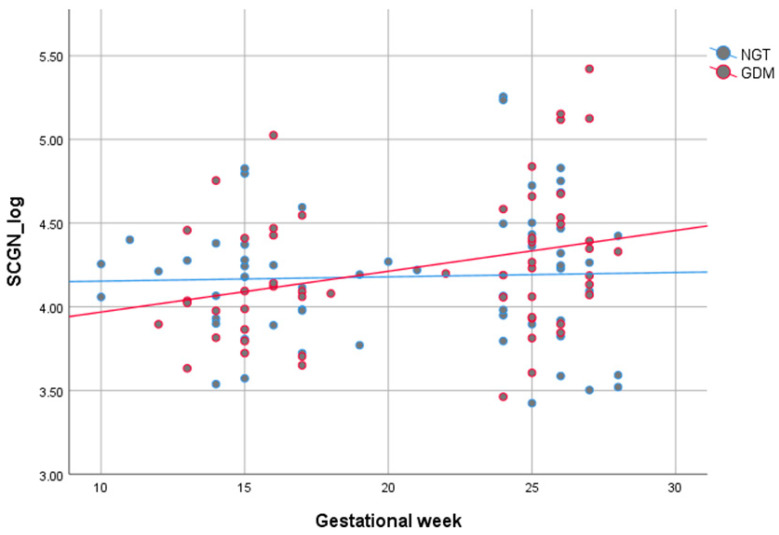
Grouped scatter dot plot according to glucose tolerance (blue = NGT, red = GDM) depicting the correlation of secretagogin (SCGN) (log-transformed) with week of gestation during pregnancy between week of gestation (GW) 10 and 28. In women with GDM, SCGN correlated positively with week of gestation (*r_p_* = 0.308, *p* = 0.021). NGT displays the same trend, albeit not statistically significant (*r_p_* = 0.035, *p* = 0.791). NGT: normal glucose tolerance; GDM: gestational diabetes mellitus.

SCGN levels increased marginally during pregnancy; SCGN levels correlated positively with GW in women with GDM (*r_p_* = 0.308, *p* = 0.021); in NGT, the correlation was not significant (see Figure 4 and Table 2).

## 4. Discussion

In the present study, SCGN was related to insulin secretion in all pregnant women, predominantly in women with NGT. SCGN correlated positively with the Stumvoll first and second phase index, which corresponds to previous studies supporting a connection between SCGN and insulin secretion. Furthermore, SCGN increased significantly postpartum compared to pregnancy. SCGN levels did not show a difference between NGT and GDM in our cohort of high-risk pregnant women in any of the visits during pregnancy and postpartum, although SCGN was elevated in type 2 diabetes mellitus patients in a previous study [14]. However, the cohort (NGT and GDM) is homogeneous in respect to GDM risk factors such as age and BMI. Furthermore, pregnancy is per se associated with increasing insulin resistance and insulin secretion [15,16]. The status of GDM, in contrast to type 2 diabetes mellitus, might, thus, not be the determining factor for differences in SCGN levels. To our best knowledge, no evaluation in pregnancy or of women with gestational diabetes mellitus has been done until now.

With regard to pancreatic islet cells, secretagogin has so far been related to insulin release in loss-of-function studies and the regulation of beta-cell proliferation [2,9,12,33]. SCGN binds insulin and improves insulin signaling when compared to insulin action alone and, accordingly, dropped over the course of an OGTT in one study [13]. We were not able to replicate these results in our OGTTs, most probably due to the low number of patients for whom these values were available. SCGN’s involvement in insulin metabolism was further supported by research on SCGN knock-out mice, which demonstrated progressing glucose intolerance most likely due to loss of beta-cell mass [2] and in vivo studies showing differences in SCGN levels between type 2 diabetes mellitus patients and controls [14]. Recent studies came to the conclusion that type 2 diabetes mellitus could be a state of SCGN deficiency [2,34,35]. Exact mechanisms remain hypothetical at this point, especially considering the heterogeneity in insulin secretion of pre-diabetes and type 2 diabetes mellitus, ranging from hyperinsulinemia to varying degrees of beta-cell dysfunction [36].

SCGN might play a different role in insulin secretion in patients with type 2 diabetes than in pregnancy and women with gestational diabetes. SCGN levels did not differ between women with GDM and NGT in the present cohort. However, SCGN is intrinsically linked to insulin secretion and involved in weight control [1,13]. The pregnant women in both groups displayed similar characteristics in terms of BMI and age. Moreover, pregnancy is a state of hyperinsulinemia and insulin resistance [15,16]. Due to these similarities between NGT and GDM, the diagnosis GDM might not be reflected in SCGN levels.

SCGN levels were significantly higher postpartum than during pregnancy. This trend was visible in both women with NGT and GDM, albeit only significant in NGT. SCGN’s ability to bind insulin [13] might offer an explanation for the lower values in pregnancy compared to postpartum due to the pregnancy-related hyperinsulinemia. Pregnancy is a state of progressing insulin resistance and hyperinsulinemia to supply the fetus with glucose [15,16]. Accordingly, SCGN correlated negatively with the Matsuda Index in women with NGT, and SCGN levels increased marginally during pregnancy in all pregnant women in this cohort. Furthermore, as SCGN is critical in neuronal growth and brain development [37], it might be involved in fetal neural development. Hypothetically, if SCGN was transferred via the maternal placenta to the fetus, it could explain the lower levels in mothers during pregnancy. However, the possibility of placental transfer is speculative at this point as SCGN has, to the best of our knowledge, not been investigated in the context of pregnancy before. Another potential explanation for the discrepancy between pregnancy and postpartum is changes in maternal kidney clearance during pregnancy [38]. These physiological adaptions might impact and heighten the renal clearance of SCGN. In support of this theory, SCGN correlated positively with creatine during pregnancy in our cohort. A noteworthy observation is the positive correlation of triglyceride levels with SCGN in women with GDM, which might indicate a compensatory mechanism to reduce triglyceride content. Accordingly, SCGN treatment reduced triglycerides and cholesterol in high-fat diet fed mice [13].

A limitation of this study is that postpartum SCGN values were not available for all women due to a high postpartum drop-out rate. Therefore, the assessment of changes from pregnancy to postpartum was limited to a restricted number of patients. Moreover, the conclusions on the relationship between insulin secretion and SCGN is based on correlations rather than causation.

Being the first study on SCGN in pregnancy and GDM, we were able to demonstrate a potential connection of SCGN to insulin secretion in pregnancy. SCGN was predominantly associated with insulin secretion in women with NGT, which suggests an involvement in the physiological increase in insulin secretion during gestation. It remains unclear whether the lower SCGN levels in pregnancy compared to postpartum are the actuator or the result of pregnancy-related changes in insulin metabolism. A larger cohort, non-pregnant controls and the addition of pre-pregnancy SCGN levels in future studies would allow for a better judgement on changes during gestation and postpartum. The discrepancy between studies in patients with type 2 diabetes mellitus compared to GDM indicate the necessity for further research. To ameliorate the understanding of the functions of this biomarker, studies in other models of insulin resistance and changing insulin secretion are imperative, for instance, in patients with impaired glucose tolerance or normal lean pregnancies.

## Figures and Tables

**Table 1:** Baseline characteristics and glycemic and metabolic parameters of oral glucose tolerance tests (OGTT) at GW 10–28 and postpartum for NGT and GDM.

	All	NGT	GDM	
	Mean (± SD)	Mean (± SD)	Mean (± SD)	*p*-Values
**Maternal Indices at GW 10–28**				
Number	93	48	45	
GDM in previous pregnancy	48.8%	40.9%	57.9%	0.094
Birth weight > 4000 g in previous pregnancy	23.5%	20.5%	27.0%	0.486
BMI before pregnancy (in kg/m²)	28.59 (± 7.06)	27.88 (± 6.69)	29.34 (± 7.44)	0.243
Waist (in cm)	114.8 (± 10.8)	114.7 (± 9.1)	115.0 (± 12.4)	0.795
Hip (in cm)	123.6 (± 11.5)	124.1 (± 8.7)	123.0 (± 14.0)	0.833
Age (in years)	33 (± 5)	32 (± 5)	33 (± 5)	0.632
Blood pressure systolic (in mmHg)	114 (± 11)	114 (± 10)	114 (± 12)	0.809
Blood pressure diastolic (in mmHg)	69 (± 8)	68 (± 8)	70 (± 9)	0.177
**Pregnancy (GW 10–28)**				
SCGN (in pg/mL)	75.24 (± 37.90)	71.57 (± 34.47)	79.15 (± 41.28)	0.514
AUC insulin	70.36 (± 45.96)	59.77 (± 33.87)	85.06 (± 56.12)	0.024
AUC glucose	131.43 (± 29.26)	110.60 (± 12.85)	160.32 (± 19.18)	<0.001
Matsuda Index	5.77 (± 4.13)	6.68 (± 4.20)	4.49 (± 3.73)	0.013
Stumvoll first phase index (in pmol/L)	1171.65 (± 605.86)	1307.64 (± 626.24)	974.24 (± 523.90)	0.003
Stumvoll second phase index (in pmol/L)	343.24 (± 159.76)	371.18 (± 168.12)	302.68 (± 139.57)	0.020
Disposition Index	5.79 (± 5.30)	7.88 (± 5.75)	2.57 (± 1.89)	<0.001
ISSI-2	292.85 (± 127.09)	354.40 (± 111.32)	207.47 (± 94.81)	<0.001
HbA1c (in mmol/mol Hb)	32.22 (± 2.6)	30.03 (± 1.9)	33.32 (± 3.24)	0.002
Triglycerides (in mg/dL)	170 (± 58)	153 (± 55)	188 (± 56)	0.014
Cholesterol (in mg/dL)	224 (± 45)	221 (± 43)	228 (± 47)	0.667
Bioavailable estradiol (pg/mL)	1231 (± 685)	1117 (± 659)	1350 (± 698)	0.100
**Postpartum**				
Number	34	19	15	
SCGN (in pg/mL)	88.07 (± 35.63)	83.88 (± 24.65)	93.37 (± 46.45)	0.683
AUC insulin	39.98 (± 27.15)	29.09 (± 11.76)	44.34 (± 30.73)	0.363
AUC glucose	118.04 (± 18.01)	111.13 (± 19.46)	120.81 (± 17.67)	0.384
Matsuda Index	8.92 (± 5.89)	10.33 (± 5.65)	8.35 (± 6.18)	0.591
Stumvoll first phase index (in pmol/L)	862.12 (± 488.50)	883.31 (± 615.93)	853.65 (± 466.86)	0.923
Stumvoll second phase index (in pmol/L)	259.71 (± 129.43)	260.98 (± 154.27)	259.20 (± 127.52)	0.983
Disposition Index	4.70 (± 2.36)	6.41 (± 3.06)	3.94 (± 1.66)	0.080
ISSI-2	273.65 (± 99.19)	304.15 (± 135.65)	261.46 (± 86.60)	0.489
HbA1c (in mmol/mol Hb)	32.24 (± 1.9)	34.41 (± 1.9)	32.24 (± 1.9)	0.157
Triglycerides (in mg/dL)	90 (± 45)	84 (± 44)	97 (± 47)	0.389
Cholesterol (in mg/dL)	193 (± 34)	189 (± 38)	197 (± 29)	0.466
Bioavailable estradiol (in pg/mL)	39 (± 55)	49 (± 69)	26 (± 22)	0.228

Continuous variables were summarized by mean ± standard deviation (SD) and categorical variables by counts and percentages. To assess differences between NGT and GDM and GDM subgroups, a T-Test was performed. NGT: normal glucose tolerance; GDM: gestational diabetes mellitus; GW: week of gestation; AUC: area under the curve; BMI: body mass index; SCGN: secretagogin; HbA1c: hemoglobin A1c; ISSI-2: insulin secretion sensitivity index.

**Table 2:** Pearson’s correlation analysis SCGN during pregnancy in all women, women with NGT and GDM. The significance level is *p* ≤ 0.05.

Pearson’ Correlation	All (in Pregnancy)		NGT (in Pregnancy)		GDM (in Pregnancy)	
	*r_p_*	*p*	*r_p_*	*p*	*r_p_*	*p*
Week of gestation	0.172	0.068	0.035	0.791	0.308	0.021
BMI of visit	−0.069	0.470	0.055	0.686	−0.174	0.203
Blood pressure systolic	0.193	0.043	0.221	0.105	0.172	0.210
Blood pressure diastolic	0.121	0.209	0.219	0.108	0.030	0.829
Matsuda Index	−0.104	0.320	−0.273	0.050	0.093	0.563
Stumvoll first phase index	0.220	0.032	0.390	0.004	−0.056	0.724
Stumvoll second phase index	0.224	0.028	0.395	0.003	−0.058	0.714
Disposition index	0.107	0.316	0.002	0.987	0.316	0.057
ISSI-2	0.082	0.436	−0.026	0.856	0.163	0.310
AUC insulin	0.132	0.206	0.380	0.005	−0.028	0.861
AUC glucose	−0.029	0.782	0.019	0.893	0.035	0.827
HbA1c	−0.158	0.092	−0.391	0.002	−0.028	0.836
Triglycerides	0.171	0.067	0.041	0.761	0.276	0.038
Cholesterol	0.169	0.072	0.078	0.560	0.246	0.065
Creatine	0.194	0.012	0.191	0.068	0.223	0.057
Bioavailable estradiol	0.139	0.139	0.151	0.263	0.113	0.402

SGGN: secretagogin; NGT: normal glucose tolerance; GDM: gestational diabetes mellitus; AUC: area under the curve; BMI: body mass index; ISSI-2: insulin secretion sensitivity index; HbA1c: hemoglobin A1c.
